# The Effect of Aerobic Exercise on Post-concussion Recovery in Athletes: A Narrative Review

**DOI:** 10.7759/cureus.100939

**Published:** 2026-01-06

**Authors:** Chloe E Esch, Garrett J Rutt, Emily Lewandowski, Marcia Ballantyne

**Affiliations:** 1 Physical Medicine and Rehabilitation, Lake Erie College of Osteopathic Medicine, Bradenton, USA; 2 Orthopedic Surgery, Lake Erie College of Osteopathic Medicine, Bradenton, USA; 3 Physical Medicine and Rehabilitation, Lake Erie College of Osteopathic Medicine, East Aurora, USA; 4 Pathology, Lake Erie College of Osteopathic Medicine, Bradenton, USA

**Keywords:** aerobic exercise, athletes, concussion, holistic health, recovery, sports-related concussion

## Abstract

Traumatic brain injuries (TBIs), in particular sports-related concussions (SRCs), are a significant health concern for young athletes in the United States. Historically, treatment has consisted of prolonged physical and cognitive rest, requiring athletes to abstain from activity until symptoms resolve and physician clearance is obtained. However, emerging evidence suggests that the early introduction of light aerobic exercise during the post-concussive period may shorten symptom duration. While several individual studies have explored this approach, no recent comprehensive literature reviews have summarized these findings.

This review examines four peer-reviewed studies, including cohort studies and randomized controlled trials, that assess the impact of light aerobic exercise on recovery in athletes diagnosed with SRCs. Articles were collected following a systematic PubMed and Google Scholar search with defined inclusion and exclusion criteria.

All studies included in this review reported faster symptom resolution for participants who engaged in light aerobic activity compared to those who followed standard rest protocols.

This review showcases the potential benefits of safely introducing light aerobic exercise into the treatment of SRC in athletes. Despite limitations such as small sample sizes and narrow age ranges, the findings support a shift in SRC management toward active rehabilitation.

## Introduction and background

It is estimated that about 1.5 million traumatic brain injuries (TBIs) occur each year in the United States, with associated medical costs exceeding $17 billion [1]. In a large survey of U.S. adolescents (grades 8, 10, and 12), 19.5% reported having at least one diagnosed concussion during their lifetime, and 5.5% reported more than one [2]. Furthermore, 70% of emergency department visits for sports- and recreation-related TBIs and concussions are among children aged 17 and younger [3]. The heightened prevalence in this age group may reflect both increased exposure and physiologic factors such as weaker cervical musculature and ongoing neurodevelopment during adolescence. The Berlin Consensus Statement notes that children and adolescents have a “prolonged physiological recovery” compared with adults, likely due to immature neural networks, developing cervical musculature, and a greater head-to-body ratio, all of which increase concussion susceptibility and symptom burden [4].

A concussion, as defined by the American Association of Neurological Surgeons (AANS), is an injury to the brain that results in a temporary loss of normal brain function due to mechanical force or trauma. Loss of consciousness is sometimes seen, but it is not required. Other symptoms include dizziness, visual disturbances, confusion, headache, disorientation, and unsteadiness [5]. Approximately 90% of concussion symptoms resolve within the post-concussive 10-14 day period; however, some symptoms may persist for more than three months [1]. At the cellular level, concussion triggers a neurometabolic cascade characterized by ionic flux, axonal dysfunction, delayed neurotransmission, inflammation, and impaired energy metabolism. This “energy crisis” renders the brain particularly vulnerable to additional injury if appropriate recovery protocols are not followed. This risk of repeated injury is especially relevant in sports-related concussions (SRCs), historically leading clinicians to recommend strict physical and cognitive rest [6]. Within this evolving understanding of concussion pathophysiology, early light aerobic exercise has been discussed as a potential means of supporting physiologic recovery during this metabolically vulnerable period.

Beyond the physical symptoms, concussion also disrupts cognitive processes, including attention, memory, processing speed, and executive function, which are particularly important for school-aged athletes who must balance academics and recovery [4,7].

Although strict rest was traditionally recommended, emerging evidence suggests that prolonged physical and cognitive inactivity may actually worsen symptoms, increase emotional distress, and delay recovery [8]. Traditional concussion protocol consists of rest with limited activity to avoid a spike in PaCO₂ with a resultant increase in cerebral blood flow and exacerbation of concussive symptoms [9]. Recent research has shown that implementing early, low-intensity aerobic exercise such as walking and biking can be more beneficial than physical and mental rest [9]. The American Academy of Pediatrics currently recommends “early return to light physical activity” rather than prolonged strict rest [10].

Given the evolution of clinical guidelines and emerging evidence questioning the benefits of prolonged rest, this review examines whether early, light aerobic exercise can safely accelerate recovery in young athletes with sport-related concussion. In this literature review, we present an up-to-date analysis of the current treatment of athletes with light aerobic exercise in the post-concussive period.

## Review

Methodology

This narrative review aimed to summarize current evidence on the role of early, low-intensity aerobic exercise in the recovery of athletes with sport-related concussion. To identify relevant literature, a broad search of PubMed and Google Scholar was conducted. Key terms included “concussion,” “sport-related concussion,” “aerobic exercise,” “recovery,” and “athlete.” Additional articles were identified by examining reference lists of key studies and relevant review papers.

The search focused primarily on recent publications from 2019 to 2022 to reflect current trends in concussion management, with emphasis on clinical trials and observational studies that examined early physical activity following concussion. Articles were selected based on their relevance to the topic and their contribution to understanding how sub-symptom threshold aerobic exercise influences recovery trajectories in youth and adolescent athletes.

Because this is a narrative review, no formal inclusion or exclusion criteria were applied, and studies were not assessed using structured risk-of-bias tools. Instead, study quality was evaluated based on key methodological features, including study design, sample characteristics, clarity of concussion diagnosis, description of the exercise intervention, outcome measures, and transparency of reporting. The literature was synthesized qualitatively, with findings organized by study design and the reported effects of aerobic exercise on symptom resolution.

Results

Four studies investigated the effect of early sub-symptom threshold aerobic exercise on SRC recovery (Table 1). 

**Table 1:** Early sub-symptom threshold aerobic exercise and sport-related concussion recovery *Hutchinson reports estimated days to medical clearance, while the other studies report days to asymptomatic status

Study	Design	Participants	Intervention	Control	Recovery Time (Intervention)	Recovery Time (Control)
Leddy et al., 2019 [11]	Randomized clinical trial	103	Sub-symptom threshold aerobic exercise	Light stretching	13 days	17 days
Wilson et al., 2020 [12]	Retrospective cohort study	575	Early physical activity (various intensities)	No activity	10.5 days	16 days
Leddy et al., 2021 [13]	Randomized clinical trial	118	Sub-symptom threshold aerobic exercise	Placebo stretching	12 days	21 days
Hutchinson et al., 2022 [14]	Randomized non-blinded clinical trial	38	Structured aerobic exercise protocol (SAEP)	Usual care exercise prescription (UCEP)	38 days*	60 days*

In a randomized clinical trial, Leddy et al. assigned 103 participants in the post-concussive period to either an aerobic exercise group or a light stretching control group [11]. The exercise group was assigned daily aerobic sub-symptom threshold exercise guided by heart-rate monitoring. Participants were monitored weekly until recovery or until the study ended after four weeks. Recovery was defined as an asymptomatic status. It was found that the exercise group recovered in a median of 13 days versus 17 days in the control group.

Wilson et al. retrospectively evaluated 575 pediatric patients with SRC, 12% of whom engaged in early post-concussion activity ranging from light aerobic exercise to non-contact training [12]. Symptom resolution occurred significantly faster in the early activity group, with a median of 10.5 days compared to the no-activity group, which had a median of 16 days.

A second randomized clinical trial by Leddy et al. recruited 118 adolescents to confirm the safety and efficacy of early aerobic exercise [13]. The aerobic group achieved clinical recovery in a median of 12 days, compared to 21 days in the stretching group. Persistent post-concussive symptoms occurred in 9% of the aerobic group versus 31% in controls.

Most recently, a longitudinal, randomized, non-blinded clinical trial of 38 participants examined the effect of a structured aerobic exercise protocol (SAEP) compared with the usual care exercise prescription (UCEP) [14]. By day 28, when the trial ended, 74% of the SAEP group were asymptomatic versus 50% in the UCEP group. Survival modeling estimated the mean time to medical clearance at 38 days for SAEP versus 60 days for UCEP.

Across the four studies, aerobic exercise post-concussion accelerated the recovery process by four to seven days. Groups that participated in sub-symptom threshold exercise recovered 35-40% faster than groups that stretched or abstained from physical activity. These findings suggest that early aerobic exercise following a concussion can be a safe and effective intervention.

Discussion

This review summarizes the results of multiple studies examining differences in recovery following SRCs when exposed to early, light aerobic exercise compared with traditional mental and physical rest [11-14]. After analyzing the data, this review demonstrates that early, light aerobic exercise performed at sub-symptom thresholds can accelerate recovery following SRCs, particularly in pediatric and adolescent populations. Prolonged symptom duration increases the risk of developing persistent post-concussive symptoms (PPCS), defined as concussion symptoms that last beyond 28 days in children and adolescents [13]. This is why prompt recovery is so crucial. Approximately one-third of pediatric patients experience PPCS following a concussion [15]. DSM-IV describes postconcussion syndrome (PCS) as the presence of cognitive deficits in attention and memory accompanied by at least three additional symptoms such as fatigue, sleep disturbance, headache, dizziness, irritability, affective disturbance, or apathy/personality change [1]. Current literature prefers to use “post-concussive symptoms” instead of implying that prolonged symptoms are a syndrome. However, the PCS definition highlights the long-term effects of lingering concussion symptoms. Prolonged concussion symptoms can also lead to epilepsy, psychological consequences, long-term memory issues, and movement disorders [16].

Young athletes often intertwine their ability to compete in sports with their mental health and overall sense of well-being. Concussions are not only disruptive to physical health but also impact mental health because they keep children and adolescents out of school and social activities [17]. Prolonged inactivity and isolation can lead to feelings of depression and an increased risk of suicidal behaviors [18]. Prescribing athletes sub-symptom threshold activity provides an opportunity for them to feel a sense of control over the situation, rather than being told to strictly rest and isolate themselves. Engaging in physical activity provides a structured and manageable way for athletes to remain connected to their recovery process. This can support emotional well-being and reduce the risk of depression or anxiety during the post-concussive period. These considerations highlight that early, guided aerobic exercise supports not only symptom resolution but also functional recovery, participation in daily activities, and psychosocial well-being.

The studies reviewed also support the safety of light aerobic exercise when guided by physiological monitoring. These methods help ensure that activity does not exacerbate injury, allowing athletes to experience the psychological and physiological benefits of exercise. These findings suggest that structured aerobic exercise could help bridge the gap between initial rest and full return to sport, potentially reducing the incidence of PPCS and minimizing academic and social disruptions.

Despite these findings, several limitations must be acknowledged. Most studies focused on pediatric or adolescent athletes, so it is unclear whether these findings also apply to adults. Sample sizes were relatively small, and follow-up durations were limited, with some studies ending at 28 days despite a subset of participants remaining symptomatic. It can also be argued that participants who engaged in physical activity did so because their symptoms were milder at baseline, allowing them to better tolerate exercise [19]. Additionally, long-term outcomes and potential delayed complications of early aerobic exercise were not evaluated. Future research should include larger, more diverse populations, extended follow-up periods, and standardized exercise protocols to validate this approach across all age groups.

## Conclusions

The shift toward integrating early, light aerobic exercise in concussion management reflects a growing emphasis on active rehabilitation strategies, supported by multiple clinical studies demonstrating its safety and efficacy in accelerating recovery. To our knowledge, this review presents the most current and comprehensive comparison of concussion management and highlights the potential benefits of integrating light aerobic exercise into sports-related concussion recovery plans for young athletes. While future research is needed to assess the applicability and generalizability of these findings, this approach appears to promote faster recovery and may help mitigate the negative effects of academic and social isolation on overall well-being. Allowing athletes to engage in controlled, light aerobic activity during recovery can provide them with a sense of autonomy, which helps them preserve their athletic identity and gives them a sense of purpose. Heart rate-guided aerobic protocols are a beneficial alternative to traditional rest following an SRC. Using exercise as a recovery method is effective and physically and emotionally safer for pediatric and adolescent athletes. The growing clinical adoption of early, guided aerobic exercise highlights its increasing acceptance as a standard component of concussion rehabilitation.
